# Infraorbital Rejuvenation Combined with Thread-Lifting and Non-cross-linked Hyaluronic Acid Injection: A Retrospective, Case-Series Study

**DOI:** 10.1007/s00266-023-03740-1

**Published:** 2023-11-14

**Authors:** Zhi-Feng Liao, Wei Yang, Xin Li, Shi-wei Wang, Fang-Cen Liu, Sheng-Kang Luo

**Affiliations:** 1 Department of Plastic and Reconstructive Surgery, Guangdong Second Provincial General Hospital, 466 Middle Xin Gang Road, Guangzhou City, 510317 Guangdong Province China; 2 Department of Medical Cosmetology, Beijing Huaxia Medical Beauty Hospital, Beijing, China; 3 Department of Medical, Imeik Technology Development Co., Ltd., Beijing, China; 4 Guangzhou Yestar Medical Aesthetic Hospital, Guangzhou City, Guangdong Province China

**Keywords:** Infraorbital rejuvenation, Hyaluronic acid injections, Thread-lifting, Infraorbital aging

## Abstract

**Background:**

Infraorbital aging develops during the natural aging process. Various treatment options offer unique benefits, accompanied by diverse side effect profiles, and can be synergistically combined to optimize results. This study aimed to evaluate the efficacy of a comprehensive approach involving non-cross-linked hyaluronic acid injection and smooth absorbable PPDO (poly *p*-dioxanone) thread insertion for infraorbital rejuvenation.

**Methods:**

This retrospective case series study enrolled ten female patients with infraorbital aging from March 2022 to April 2023. Clinical outcomes, patient satisfaction, and adverse events were assessed at 1, 3, and 6 months posttreatment.

**Results:**

The median Global Aesthetic Improvement Scale scores evaluated by the operator and blinded evaluator were 1.70 ± 0.42 and 1.80 ± 0.35, respectively, at six months posttreatment. The median Allergan Infraorbital Hollows Scale determined by the operator was 1.15 ± 0.34 at six months posttreatment, whereas the scores evaluated by the blinded evaluator were 1.15 ± 0.53. At six months after treatment, 50% of patients were satisfied, and an additional 40% reported strong satisfaction with the clinical improvement following treatment. No serious adverse events, such as infections, lumps, irregularities, Tyndall effect, hematoma, or skin necrosis, occurred during the treatment period.

**Conclusions:**

The combination of PPDO thread insertion and non-cross-linked hyaluronic acid injection yielded satisfactory and effective clinical outcomes with no occurrence of serious adverse events for infraorbital rejuvenation. We anticipate that this study will contribute to the advancement of novel treatment options for infraorbital aging.

**Level of Evidence IV:**

This journal requires that authors assign a level of evidence to each article. For a full description of these Evidence-Based Medicine ratings, please refer to the Table of Contents or the online Instructions to Authors www.springer.com/00266.

**Supplementary Information:**

The online version contains supplementary material available at 10.1007/s00266-023-03740-1.

## Background

The infraorbital area is one of the primary regions to show signs of aging, making a significant contribution to the overall facial appearance. The aging process is influenced by a combination of genetic composition and environmental factors. Infraorbital aging is characterized by the appearance of fine wrinkles, infraorbital dark circles, tear trough deformity, and infraorbital hollowing, collectively contributing to a fatigued and aged facial appearance. Consequently, infraorbital rejuvenation plays a pivotal role in enhancing the appearance of the aging face. The treatment of the infraorbital region is essential for achieving a more youthful and rejuvenated overall facial aesthetic [1–6].

Recent advancements in cosmetic medicine have contributed to the growing popularity of nonsurgical and minimally invasive procedures as the preferred treatment options. Commonly used treatments today include topical therapies, mechanical and chemical skin resurfacing techniques, lasers, radiofrequency devices, fillers, and neuromodulation through botulinum toxin. These treatment options offer unique advantages along with varying side effect profiles, and they can be combined synergistically to optimize results. As a result, patients have a diverse range of options to achieve their desired cosmetic outcomes while minimizing invasiveness and downtime [5, 7].

The main objectives of rejuvenating the area around the eyes include restoring soft tissue volume, smoothing fine wrinkles, and improving skin quality. Soft tissue filler injection has emerged as a straightforward and effective procedure to correct tear trough deformity, delivering immediate and noticeable results with a relatively short recovery time. This treatment approach addresses multiple aspects of infraorbital aging and offers patients a convenient and efficient way to achieve a more youthful and refreshed appearance around the eyes [8–10]. However, despite reports of aesthetically pleasing outcomes with filler use, infraorbital rejuvenation using soft tissue fillers can result in unnatural contours and puffiness due to the complex anatomical features in this area. Additionally, the thin skin in the periorbital region and the relative scarcity of fatty tissue increase the risk of producing lumps, irregularities, and the Tyndall effect when injecting cross-linked hyaluronic acid into the infraorbital area. As such, careful consideration of patient anatomy and selection of appropriate injection techniques are essential for achieving satisfactory and natural-looking results in infraorbital rejuvenation procedures.

Previous studies have indicated that the insertion of PDO (polydioxanone) threads can be a valuable, supplementary, and non-invasive technique for infraorbital rejuvenation [11]. Our study introduced a comprehensive approach involving the use of non-cross-linked hyaluronic acid and PPDO (Poly-*p*-dioxanone) thread inserts, which yielded satisfactory and effective clinical outcomes in treating horizontal neck wrinkles [12]. So, the aim of this study is to outline a novel approach for infraorbital rejuvenation, employing non-cross-linked hyaluronic acid injection and PPDO (poly-p-dioxanone) thread insert. The objective of thread-lifting and non-cross-linked hyaluronic acid injection is to address infraorbital hollowing and smooth fine wrinkles. Additionally, we conducted a discussion on the safety and efficacy of this innovative approach.

## Materials and Methods

### Patients

The retrospective study presented data recorded between March 2022 and April 2023. It included a total of 10 patients with infraorbital aging who underwent thread lifting using PPDO threads and non-cross-linked hyaluronic acid injection. Before enrollment, all participants provided written informed consent.

This study excluded patients with bleeding tendencies, coagulation disorders, severe diabetes, hypertension, hypertrophic or keloid scars, hypersensitivity to any of the components of injected non-cross-linked hyaluronic acid, or other systemic diseases. Additionally, patients who had undergone periorbital laser therapy, chemical peeling, botulinum toxin injections, thread implantation, soft tissue material filling, or surgery within the past 6 months were excluded from the study.

### Preoperative Preparation

Prior to the thread-lifting and non-cross-linked hyaluronic acid injection procedures, all patients underwent routine physical examinations and photographic evaluations. The markings for the procedures were made while the patient was in a sitting position. The areas to be treated were topically anesthetized using 8% lidocaine cream, applied 30–60 minutes before the treatment.

### PPDO (Poly p-dioxanon) Thread-Lifting

In this study, we used a smooth absorbable suture material called PPDO (Poly p-dioxanon) thread, which was manufactured by IMEIK Technology Development Co., Ltd. (Beijing, China). The patients were positioned in the supine position to expose the infraorbital area. Following completion of asepsis and antisepsis measures, the incision site and insertion areas received injections of local anesthesia (lidocaine 1% and adrenaline 1:200,000) to provide analgesia and hemostasis, respectively. Subsequently, the thread was inserted at a 90° angle to the skin surface, passing through the dermis at the entry point and then inserted subcutaneously. The number of threads placed was based on the extent of infraorbital hollowing present in each patient.

### Injection of Non-Cross-Linked HA

**Fig. 1:**
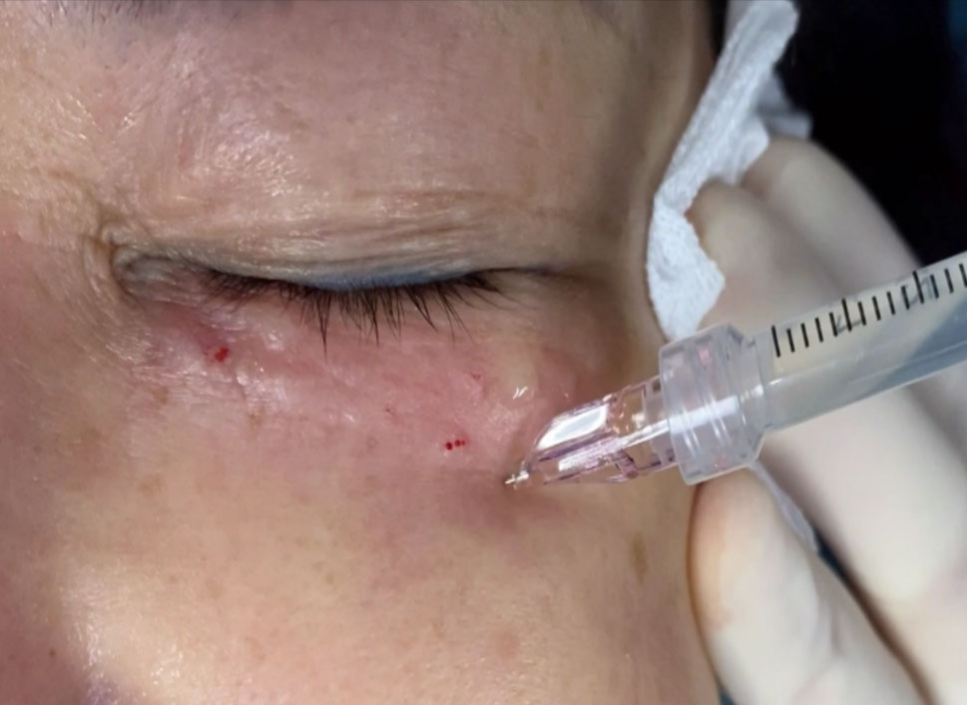
Schematic view of non-cross-linked hyaluronic acid injection

The hyaluronic acid (HA) injection was administered immediately after the completion of the thread-lifting procedure. The filler used in this study, named "Hearty" and manufactured by IMEIK Technology Development Co., Ltd., was a sodium hyaluronate composite solution suitable for injection. It comprised non-cross-linked hyaluronic acid, amino acids, vitamins, and other components [13]. A 34-gauge needle was fully inserted into the infraorbital area at an insertion angle of 10 to 15 degrees to the skin surface, with the bevel facing upward. (Figure 1) The injections were administered into the intradermal layer of the infraorbital area, and the needle was withdrawn using the linear threading technique. Each puncture received a volume of 0.01–0.02 mL of filler solution injected into the reticular dermis. The amount of filler used varied based on the depth, length, and number of wrinkles unique to each patient. Following the injections, gentle massage and pressure were applied to the injection sites. The micro-injection therapy protocol consisted of four sessions with a 4-week interval between each session. The follow-up period lasted for 6 months.

The average duration of the procedures ranged from 60 to 90 minutes. Following the completion of the combined treatment, ice packs were applied to the treated area to reduce swelling and edema. Patients were instructed to refrain from engaging in vigorous exercises for a period of seven days. Patients were prescribed Melilotus extract tablets (100mg) to be taken three times a day for three days. Prophylactic administration of antibiotics was not performed for the patients.

### Objective and Subjective Clinical Assessment

Photographs were taken in anterior view at the baseline examination (pre-injection) and 1, 3, 6 months posttreatment. Each subject was photographed under standard conditions that included the same photographer, consistent camera settings, standing posture, and uniform lighting.

#### Global Aesthetic Improvement Scale (GAIS)

After one treatment of thread lifting and four treatments of non-cross-linked HA compound, the operator and another plastic surgeon who had no conflicts and did not participate in the study procedures objectively evaluated the overall improvement of the patients’ infraorbital aging. Specifically, they each analyzed the pictures of the patients before and after treatment and carried out their assessments based on the Global Aesthetic Improvement Scale (1-very much improved, 2-much improved, 3-improved, 4-no change, 5-worse) [14].

### Allergan Infraorbital Hollows Scale (AIHS)

The operator and another independent surgeon were asked to assess the infraorbital aging of patient, based on Allergan Infraorbital Hollows Scale, which was done at baseline, 1, 3, and 6 months postoperatively (Table 1) [15].

**Table 1:** Allergan infraorbital hollows scale descriptors

Grade	Term	Description
0	None	No visible hollowing or volume loss medially or laterally
1	Minimal	Presence of hollowing with some volume loss medial to the midpupillary line; smooth lateral lid–cheek transition
2	Moderate	Defined hollowing extending laterally beyond the midpupillary line with moderate volume loss; smooth lateral lid–cheek transition with mild volume loss
3	Severe	Defined hollowing extending laterally beyond the midpupillary line with moderate volume loss creating a defined groove along the lid–cheek junction
4	Extreme	Defined hollowing extends from medial to lateral canthus; severe volume loss creates a marked step along the lid–cheek junction

### Patient Satisfaction

Patients’ satisfaction at 1, 3, and 6 months posttreatment was evaluated using the 13-item patient satisfaction questionnaire that was graded using a five-point Likert response scale (1 = strongly agree, 2 = agree, 3 = neither agree nor disagree, 4 = disagree, and 5 = strongly disagree).

### Safe Assessment

Throughout the study, participants were required to report any adverse symptoms they experienced and record the duration of these events. Safety evaluations included all abnormal reactions, including local reactions on the facial area, that occurred during the clinical test. Safety was assessed through physical examinations conducted during the clinical test period. All abnormal reactions were carefully documented. Patients' discomfort encompassed the presence and persistence of edema, bruises, palpable nodule-like lesions, and dynamic discomfort during smiling.

### Statistical Analysis

Data analysis was conducted using SPSS version 22.0 (IBM Corporation). Mean ± standard deviation values were used to present quantitative variables, such as baseline information. A *t*-test was performed to compare two physician's global assessment scores and Allergan infraorbital hollows scale, with *p*< 0.05 indicating statistical significance

## Results

### Efficacy

**Fig. 2:**
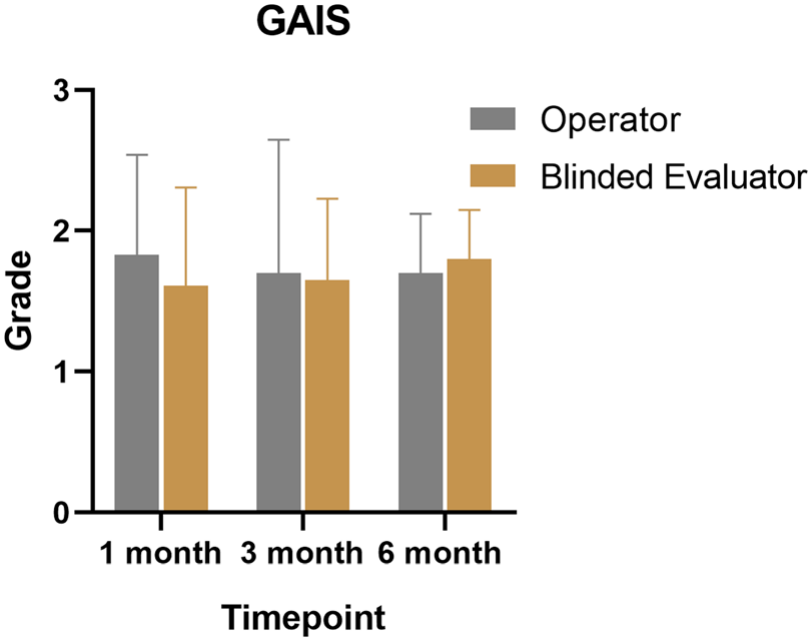
Global Aesthetic Improvement Scale was obtained by the operator and blinded evaluator at 1, 3, and 6 months post-treatment

The median age of the patients was 46.7 ± 8.6 years. The clinical outcomes were objectively assessed using the GAIS and AIHS. The median GAIS determined by the operator was 1.70 ± 0.42 at six months post-treatment, while the median GAIS evaluated by the blinded evaluator was 1.80 ± 0.35 (Table 2, Figure 2). There was no statistically significant difference in the GAIS between the operator and blinded evaluator at 1, 3, and 6 months (*p*>0.05).

**Table 2:** Patient demographic and clinical characteristics

No. of patients	
Age	46.7 ± 8.6
*Sex*	
Male	0
Female	10
*AIHS (operator, blinded evaluator)*	
Baseline	3.20 ± 0.71, 3.10 ± 0.88
1 month	1.89 ± 0.65, 1.83 ± 0.83
3 month	1.25 ± 0.79, 1.35 ± 0.53
6 month	1.15 ± 0.34, 1.15 ± 0.53
*GAIS (operator, blinded evaluator)*	
1 month	1.83 ± 0.71, 1.61 ± 0.70
3 month	1.70 ± 0.95, 1.65 ± 0.58
6 month	1.70 ± 0.42, 1.80 ± 0.35
*Complications*	
Swelling	40%
Bruise	20%
Tyndall effect	–
Irregularities	–
Lump	–
Hematoma	–
Infection	–
Skin necrosis	–

**Fig. 3:**
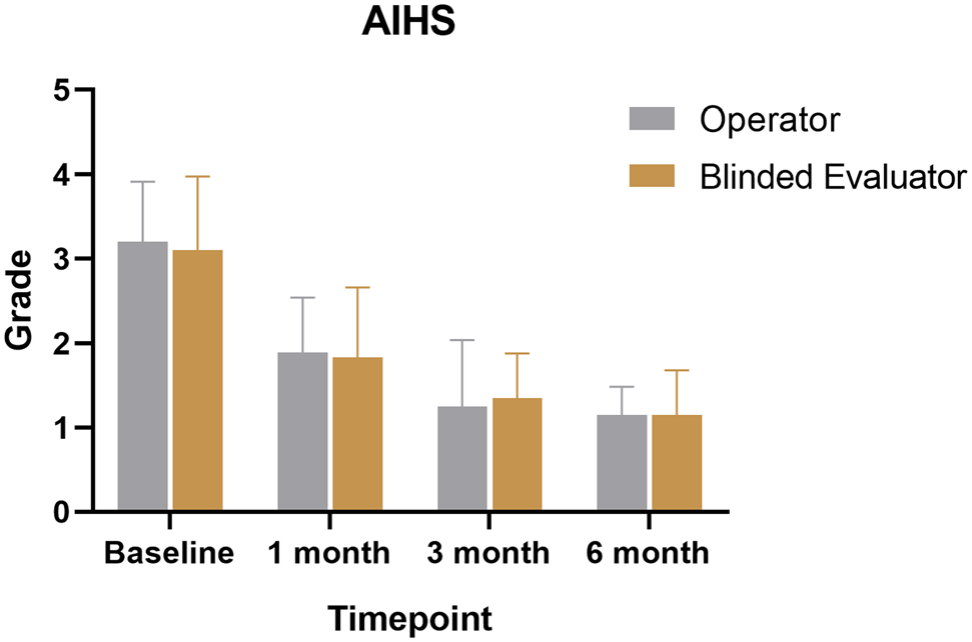
Allergan Infraorbital Hollows Scale was obtained by the operator and blinded evaluator at baseline, 1, 3, and 6 months posttreatment

The median AIHS determined by the operator was 1.15 ± 0.34 at six months post-treatment, while the median AIHS evaluated by the blinded evaluator was 1.15 ± 0.53 (Table 2, Figure 3). There was no statistically significant difference in the AIHS between the operator and blinded evaluator at 1, 3, and 6 months (*p*>0.05).

**Fig. 4:**
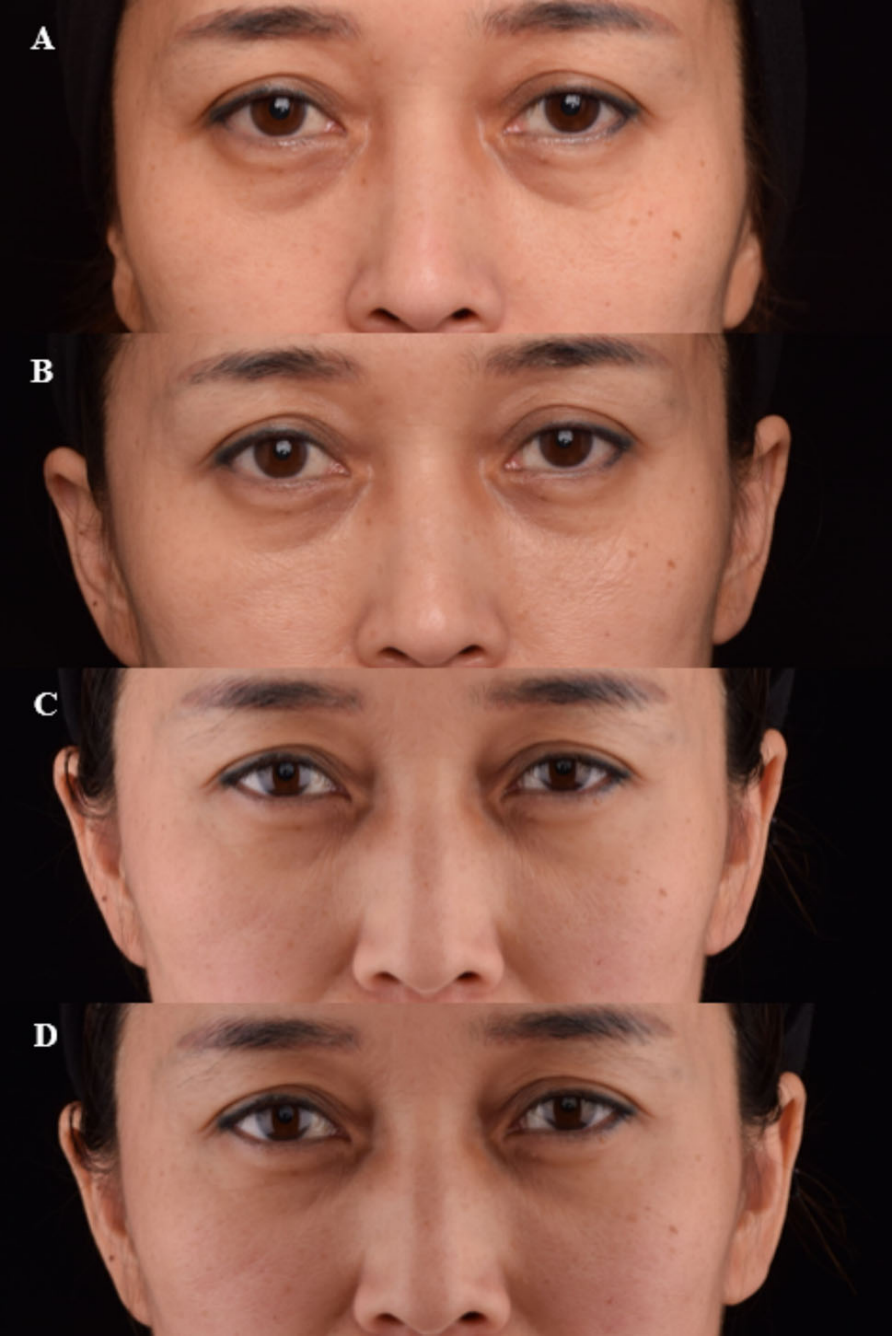
Photographs of a 50-year-old female patient: **A** before treatment **B** at 1-month follow-up **C** at 3 month follow-up **D** at 6-month follow-up

**Fig. 5:**
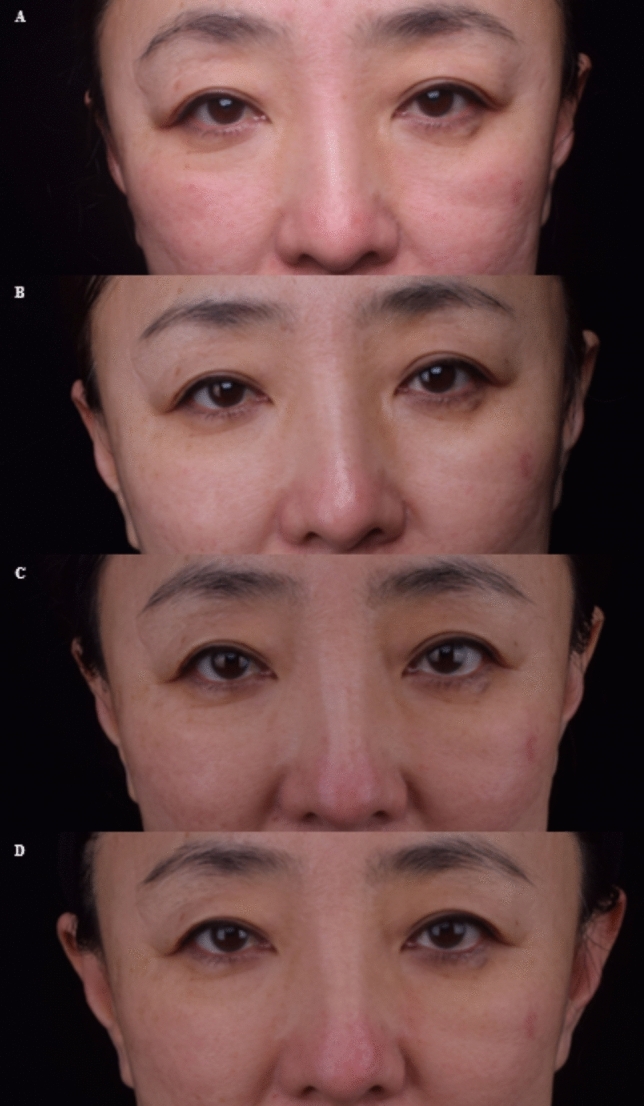
Photographs of a 59-year-old female patient: **A** before treatment **B** at 1-month follow-up **C** at 3-month follow-up **D** at 6-month follow-up

Figures 4 and 5 show the pretreatment and posttreatment images of the patients and the efficacy of the procedure.

### Patient Satisfaction

One month after treatment, 60% of patients expressed satisfaction, with 20% indicating strong satisfaction. At three months after treatment, 60% of patients were satisfied, and 30% were strongly satisfied. At six months after treatment, 50% of patients were satisfied, and 40% were strongly satisfied. The patient satisfaction rate increased with the prolongation of treatment time (Table 3).

**Table 3:** Patient satisfaction

Follow-up time	Strongly agree	Agree	Neither agree nor disagree	Disagree	Strongly disagree
1 month	2(20%)	6(60%)	2(20%)	0	0
3 month	3(30%)	6(60%)	1(10%)	0	0
6 month	4(40%)	5(50%)	1(10%)	0	0

### Safety

No serious adverse events occurred in any of the patients. Four patients experienced injection-related reactions, such as swelling, and 2 patients reported bruises, which resolved within 1 week. No adverse events occurred during the follow-up period, indicating that thread-lifting and non-cross-linked hyaluronic acid injection are safe and well-tolerated treatments for infraorbital aging (Table 2).

## Discussion

Periorbital rejuvenation offers a diverse range of treatment options. It is essential to carefully assess the severity of aging and understand patient expectations to determine the most suitable therapeutic approach. Early periorbital aging is primarily attributed to changes in the quality and quantity of soft tissue within the periorbital region, leading to infraorbital grooves and fine wrinkles [5].

In this study, we evaluated the clinical efficacy and safety of a comprehensive approach to treat infraorbital aging using HA and thread-lifting. The present data revealed that at six months posttreatment, the clinical improvement scores determined by the GAIS from the operator and blinded evaluator were 1.70 and 1.80, respectively. Besides, the AIHS showed clinical improvement of the infraorbital hollowing. (Table 2)

**Fig. 6:**
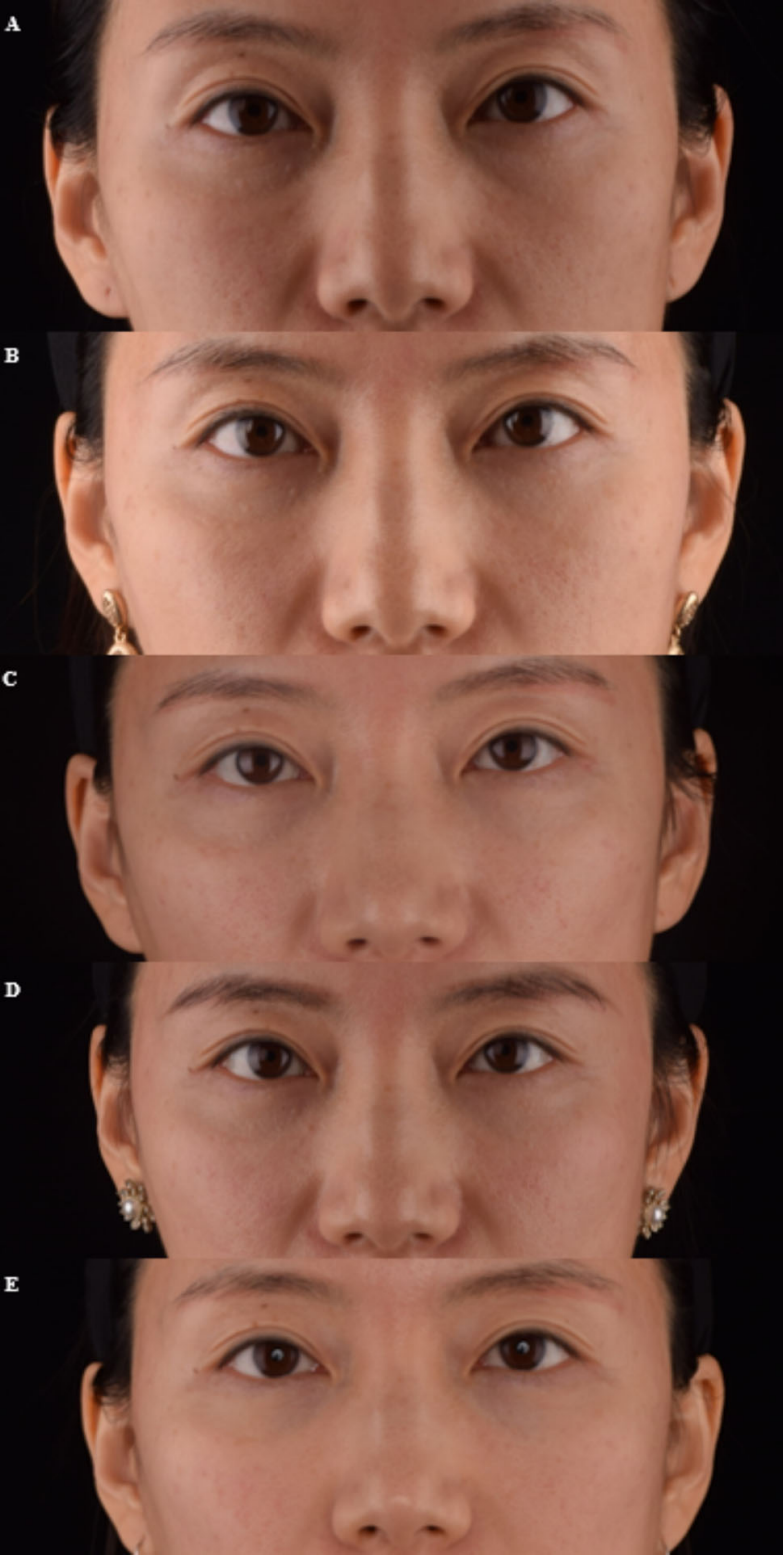
Photographs of a 47-year-old female patient: **A** before treatment **B** at 1-month follow-up **C** at 3-month follow-up **D** at 6-month follow-up **E** at 12-month follow-up

**Fig. 7:**
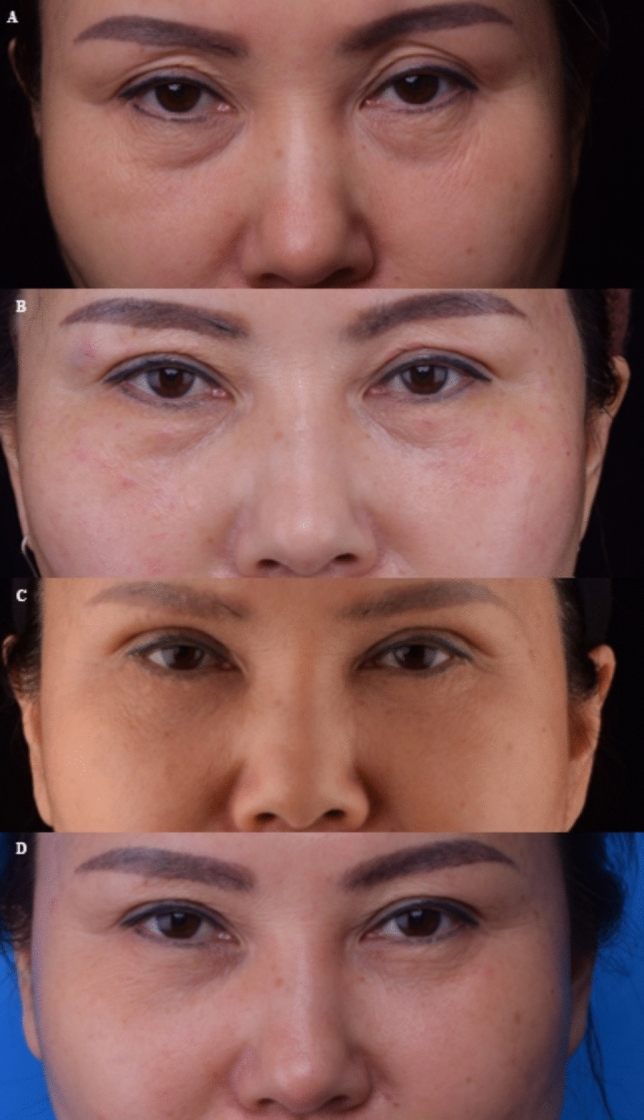
Photographs of a 53-year-old female patient: **A** before treatment **B** at 3-month follow-up **C** at 6-month follow-up **D** at 12-month follow-up

Also, all ten patients were satisfied with the improvement that occurred in their infraorbital aging at 6 months. In addition, two patients were followed for 12 month, and it showed satisfying clinical improvement at 12 month posttreatment. (Figures 6, 7) Four patients only reported mild bruising, swelling following the filler injection and thread lifting, which are widely considered the common AEs of injection and thread lifting, and the AEs lasted only for a short duration (7 days) and were resolved with a gentle ice compress. Within the six months of follow-up, no serious adverse events, including infections, lumps, irregularities, or the Tyndall effect, occurred.

The infraorbital groove consists of the nasojugal groove (also known as tear trough) medially and the palpebromalar groove laterally [16]. Various nonsurgical and surgical techniques have been proposed for correcting the infraorbital groove [17–19]. Nonsurgical rejuvenation through volumetric enhancement, involving contouring with different soft tissue fillers [20] or fat [21], has gained recognition for periorbital rejuvenation. While autologous fat serves as a suitable filler, issues such as graft absorption and the risk of irregularities are associated with fat grafting. Soft tissue filler injection is a common and convenient method for correcting the infraorbital groove, providing immediate and noticeable results with a relatively short recovery time. However, it may lead to puffiness and unnatural contours in periorbital rejuvenation using soft tissue fillers. Additionally, addressing the nasojugal area with a soft tissue filler can be challenging due to the limited overlying soft tissue. Furthermore, the angular artery and angular vein, branching from the facial artery and facial vein, respectively, are superficially located, posing a risk of vascular complications [22]. The angular artery is occasionally connected to the dorsal nasal artery, a terminal branch of the ophthalmic artery, and injury to this vessel could result in severe visual impairment. There have been reports of nasolacrimal duct obstruction and diplopia caused by filler injections [23, 24]. Hence, when performing filler injections, physicians must consider these potential complications. As an alternative approach, some surgeons use thread lifting to correct the infraorbital groove and achieve satisfactory results [11, 25]. Previous studies have shown that threads inserted into the subcutaneous tissue layer lead to immediate volume enhancement due to thread volume and induced swelling. Smooth-surfaced PPDO threads are particularly suitable for filling depressions and restoring soft tissue volume [12].

The filler used for injection in this study was a sodium hyaluronate composite solution, consisting of non-cross-linked HA, amino acids, vitamins, and other components. Injecting non-cross-linked HA into the skin yields a volume-filling effect and stimulates collagen synthesis, leading to a noticeable reduction in fine wrinkles. It effectively restores skin hydrobalance and improves skin texture, brightness, and elasticity. Non-cross-linked HA, when compared to cross-linked HA, reduces the risk of lump formation, irregularities, and vascular complications, especially in the thin periorbital skin. Previous studies, including Wang [13] and our own [12], have demonstrated the short-term efficacy and safety of non-cross-linked HA for treating mild-to-severe horizontal neck wrinkles. Additionally, due to the rapid metabolism of non-cross-linked hyaluronic acid, we conducted four treatments to sustain its therapeutic effect on the skin.

Combining non-cross-linked HA injection with smooth absorbable PPDO insertion, we believe, can effectively prevent treatment-related complications and extend the filler's efficacy by enhancing local tissue protection and repair. Giving the skin thinness and presence of vital neurovascular elements in the periocular region, any injection and or implantation of foreign bodies could potentially be associated with risks such as vascular compromise, diplopia, irregularities, lumps, etc. On the other hand, it is not clear if their patients could have had the same results with one instead of combined filler-PPDO. However, the lack of a control group made it challenging to design this study as a parallel comparative one to thoroughly investigate the functions of HA injection and PPDO thread lifting. Additionally, the study's limitations include a small patient sample size and short follow-up duration. Further studies should be conducted to investigate the long-term effectiveness of combining non-cross-linked HA injection with smooth absorbable PPDO insertion.

## Conclusion

In conclusion, our study suggests that the combined treatment of HA filler injections and thread lifting is a safe and effective approach for addressing infraorbital aging. However, to validate our findings, further studies with control groups and larger sample sizes are required.

### Supplementary Information

Below is the link to the electronic supplementary material.

Supplementary file1 (MP4 100253 KB)
